# The REASON score: an epigenetic and clinicopathologic score to predict risk of poor survival in patients with early stage oral squamous cell carcinoma

**DOI:** 10.1186/s40364-021-00292-x

**Published:** 2021-06-05

**Authors:** Chi T. Viet, Gary Yu, Kesava Asam, Carissa M. Thomas, Angela J. Yoon, Yan Chen Wongworawat, Mina Haghighiabyaneh, Courtney A. Kilkuts, Caitlyn M. McGue, Marcus A. Couey, Nicholas F. Callahan, Coleen Doan, Paul C. Walker, Khanh Nguyen, Stephanie C. Kidd, Steve C. Lee, Anupama Grandhi, Allen C. Cheng, Ashish A. Patel, Elizabeth Philipone, Olivia L. Ricks, Clint T. Allen, Bradley E. Aouizerat

**Affiliations:** 1 Department of Oral and Maxillofacial Surgery, Loma Linda University School of Dentistry, 11092 Anderson St., Suite 3304, Loma Linda, CA 92350 USA; 2 New York University Rory Meyers College of Nursing, New York, NY USA; 3 Department of Oral and Maxillofacial Surgery, New York University College of Dentistry, New York, NY USA; 4 Bluestone Center for Clinical Research, New York University College of Dentistry, New York, NY USA; 5 Department of Otolaryngology, University of Alabama at Birmingham, Birmingham, AL USA; 6 Division of Oral and Maxillofacial Pathology, Department of Pathology & Cell Biology, Columbia University College of Dental Medicine, Columbia University Irving Medical Center, New York, NY USA; 7 Department of Pathology, Loma Linda University School of Medicine, Loma Linda, CA USA; 8 Head and Neck Surgery, Providence Cancer Institute, Portland, OR USA; 9 Head and Neck Surgery, Legacy Cancer Center, Portland, OR USA; 10 Department of Oral and Maxillofacial Surgery, University of Illinois at Chicago, College of Dentistry, Chicago, IL USA; 11 Department of Otolaryngology, Loma Linda University School of Medicine, Loma Linda, CA USA; 12 School of Medicine, University of Alabama at Birmingham, Birmingham, AL USA; 13 Section on Translational Tumor Immunology, National Institute on Deafness and Other Communication Disorders (NIDCD), National Institutes of Health (NIH), Bethesda, MD USA

## Abstract

**Background:**

Oral squamous cell carcinoma (OSCC) is a capricious cancer with poor survival rates, even for early-stage patients. There is a pressing need to develop more precise risk assessment methods to appropriately tailor clinical treatment. Genome-wide association studies have not produced a viable biomarker. However, these studies are limited by using heterogeneous cohorts, not focusing on methylation although OSCC is a heavily epigenetically-regulated cancer, and not combining molecular data with clinicopathologic data for risk prediction. In this study we focused on early-stage (I/II) OSCC and created a risk score called the REASON score, which combines clinicopathologic characteristics with a 12-gene methylation signature, to predict the risk of 5-year mortality.

**Methods:**

We combined data from an internal cohort (*n* = 515) and The Cancer Genome Atlas (TCGA) cohort (*n* = 58). We collected clinicopathologic data from both cohorts to derive the non-molecular portion of the REASON score. We then analyzed the TCGA cohort DNA methylation data to derive the molecular portion of the risk score.

**Results:**

5-year disease specific survival was 63% for the internal cohort and 86% for the TCGA cohort. The clinicopathologic features with the highest predictive ability among the two the cohorts were age, race, sex, tobacco use, alcohol use, histologic grade, stage, perineural invasion (PNI), lymphovascular invasion (LVI), and margin status. This panel of 10 non-molecular features predicted 5-year mortality risk with a concordance (c)-index = 0.67. Our molecular panel consisted of a 12-gene methylation signature (i.e., *HORMAD2, MYLK, GPR133, SOX8, TRPA1, ABCA2, HGFAC, MCPH1, WDR86, CACNA1H, RNF216, CCNJL*), which had the most significant differential methylation between patients who survived vs. died by 5 years. All 12 genes have already been linked to survival in other cancers. Of the genes, only *SOX8* was previously associated with OSCC; our study was the first to link the remaining 11 genes to OSCC survival. The combined molecular and non-molecular panel formed the REASON score, which predicted risk of death with a c-index = 0.915.

**Conclusions:**

The REASON score is a promising biomarker to predict risk of mortality in early-stage OSCC patients. Validation of the REASON score in a larger independent cohort is warranted.

**Supplementary Information:**

The online version contains supplementary material available at 10.1186/s40364-021-00292-x.

## Introduction

Oral cancer is on the rise [1–3]. Each year 30,000 Americans are diagnosed with oral cavity squamous cell carcinoma (OSCC) and 80% of newly diagnosed cases are early stage I/II without regional lymph node involvement or distant metastasis. Even for early stage oral cancer patients, the five-year survival rate is as low as 60% [4–6]. The mortality rate is worse in racially and socioeconomically disadvantaged groups. A study using the Surveillance, Epidemiology, and End Results (SEER) database indicates that while black patients only make up 7.6% of all OSCC patients, with 75% of patients being white, black patients are significantly more likely to die of OSCC, which is partially a result of a later stage at diagnosis and access to healthcare or health coverage [6]. OSCC patients are treated with surgical resection of the cancer and neck lymphadenectomy, followed by adjuvant radiation with or without chemotherapy and immunotherapy based on risk stratification. However, with our current clinical practices of relying solely on clinicopathologic information, risk prediction, and therefore survival, remain poor. This poor survival rate is in contrast to other cancers, or even other head and neck cancer subtypes, such as oropharyngeal SCC, which has seen significantly improved survival, due to accurate risk stratification [7]. There is a need to develop a robust prognostic biomarker that combines both clinicopathologic data with molecular signatures to stratify OSCC patients into high and low risk categories, which will guide clinical decision making about adjuvant chemotherapy and radiation, and ultimately improve survival. OSCC is a heavily epigenetically-regulated cancer, with methylation being the most common epigenetic change; methylation leads to genomic instability and dysregulation of critical genes that enable OSCC progression [8]. Methylation is one of the most frequent events occurring early in oral carcinogenesis that is linked to cancer progression [8]. While several methylation studies in OSCC patients [8–18], including our own studies [9, 10], have highlighted specific genes controlled by methylation, none of these studies have produced a methylation biomarker with clinically meaningful prognostic ability. The main shortcomings in OSCC biomarker studies to date include: 1) combining OSCC with other head and neck cancer sub-sites (i.e.*,* oropharynyx), which creates a heterogeneous cohort, and 2) relying solely on the molecular data, without taking into account the clinicopathologic factors. In contrast, commercialized biomarkers for other cancers combine both molecular and non-molecular data to determine risk in a focused cancer subtype [19, 20]. Our current biomarker study directly addresses these shortcomings.

In this study we hypothesized that gene methylation could be combined with clinicopathologic factors to form a composite molecular and non-molecular signature with high prognostic performance in determining risk of 5-year mortality in early stage (I/II) OSCC patients. To test our hypothesis and develop our composite molecular and non-molecular risk score, we analyzed clinicopathologic data of an internal retrospective cohort of 515 OSCC patients as well as a cohort of 58 patients from The Cancer Genome Atlas (TCGA). We determined the top clinicopathologic factors that were highly predictive of 5-year disease-specific mortality in these two cohorts. We then analyzed available methylation array data in the TCGA cohort and discovered 12 genes that were differentially methylated between the OSCC patients who died by 5 years and those who survived. We combined the clinicopathologic factors with the 12-gene methylation signature into a risk score, which we named the high-Risk Epigenetic And clinicopathologic Score for Oral caNcer (REASON) score. We determined its predictive performance to identify early-stage OSCC patients who died from their disease within 5 years of diagnosis.

## Methods

### Patient selection and data collection

The patients in this study were selected from an existing OSCC database compiled at the institution at which they were treated. Collection of clinical data for this database was approved by the Institutional Review Board at each institution, which included Loma Linda University (LLU), and Columbia University Irving Medical Center (CUIMC), Portland Providence Medical Center (PPMC), University of Illinois Chicago (UIC), and University of Alabama at Birmingham (UAB). The search was limited to only oral cavity sub-sites, including oral tongue, maxillary and mandibular gingiva, hard palate, floor of mouth, buccal mucosa, and lip mucosa. Clinical and pathologic stages were recorded based on the American Joint Committee on Cancer (AJCC) Eighth Edition Staging Manual [21]. All patients had stage I or II (i.e.*,* T1N0M0 or T2N0M0) biopsy-confirmed OSCC. De-identified patient clinicopathologic characteristics were used in the data interpretation. We collected the following information from the chart review: age, sex, race, smoking and alcohol use, staging, tumor location, pathologic characteristics [i.e.*,* perineural invasion (PNI), lymphovascular invasion (LVI), margin status, histologic grade], and treatment modalities received in addition to tumor ablation (i.e.*,* neck lymphadenectomy, radiation therapy with or without chemotherapy).

### TCGA Illumina methylation array analysis

**Fig. 1:**
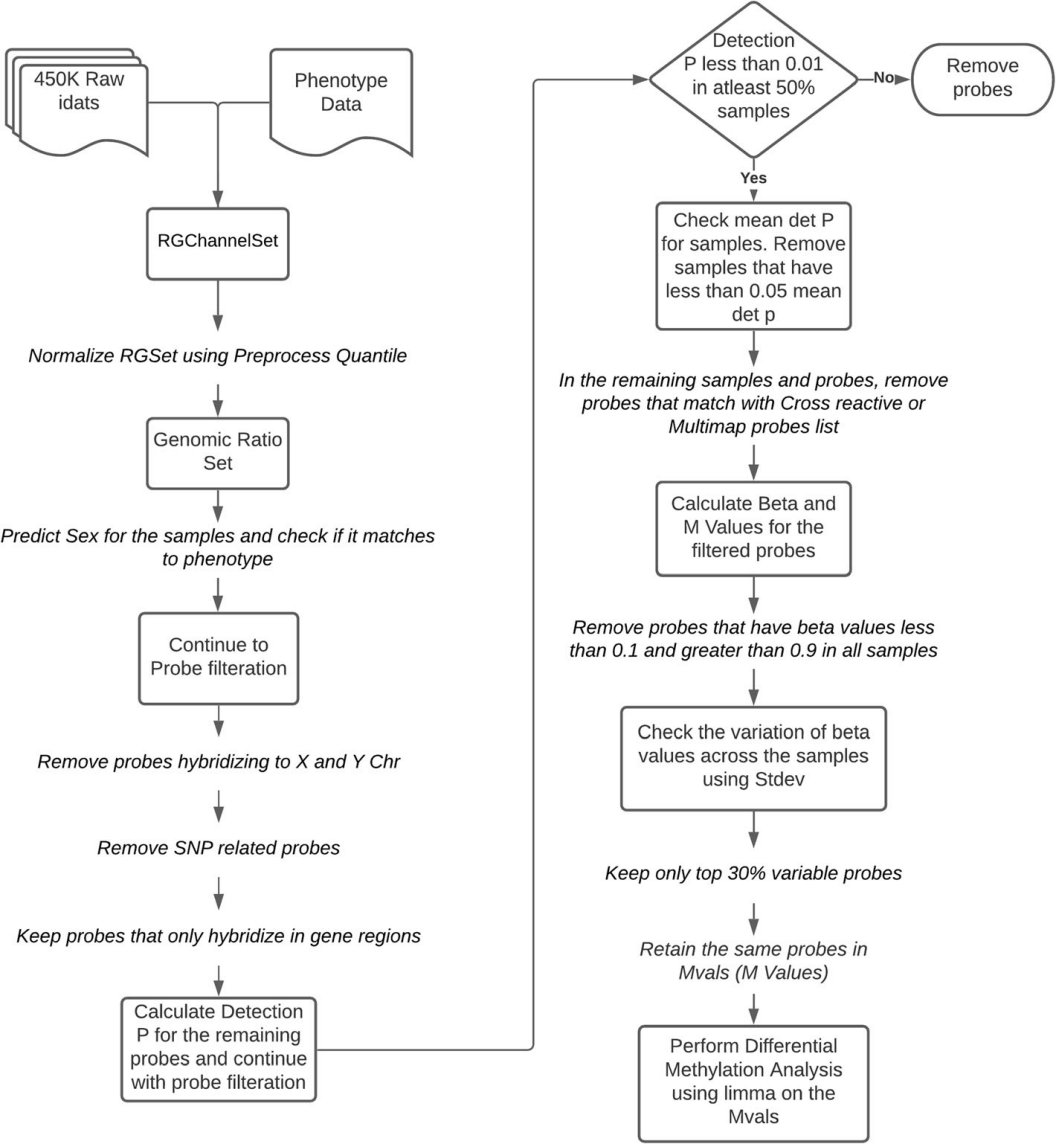
Methylation array work flow. The analysis steps for the methylation array data from the TCGA cohort are shown

We performed an analysis of methylation data from early-stage OSCC patients in the TCGA database. DNA methylation data pre-processing, quality control filtering, and quantile normalization [22] (inclusive of batch correction and surrogate variable analysis) were conducted employing the *minfi* package v1.34.0 in R [23]. Differential methylation analysis was performed using the *limma* package v3.44.3 in R [24]. The Illumina Infinium Methylation 450 K Array data analyses are outlined in the workflow in Fig. 1. Briefly, out of a total of 485,512 probes, probes that hybridized to the X or Y chromosomes were removed, leaving 473,864 probes. An additional 17,351 probes related to single nucleotide polymorphisms (SNPs) and 111,977 probes that did not map to gene regions were removed. From the remaining 344,536 probes, we retained those that had a detection *p* value of < 0.01 in at least 50% of the samples. We then filtered for probes that were cross reactive or mapped to multiple genomic positions, leaving 324,465 probes.

We excluded probes with a beta value of < 0.1 across all samples or > 0.9 across all samples, leaving 317,016 probes. Using the patient’s survival status as the outcome variable we performed batch correction using surrogate variable analysis. Surrogate variables with a correlation of higher than 0.2 with survival status were excluded (3 of 14 surrogate variables identified). We then selected the top 30% most variable methylated probes, which gave us a total number of 95,104 probes spanning 4544 genes retained for differential methylation analysis. Given the exploratory nature of this pilot study and the modest sample size available for analysis (*n* = 58), differentially methylated CpG for survival status showing an adjusted *p*-value of < 0.1 were considered for inclusion in the molecular component of the prognostic panel. Heat maps were constructed using hierarchical clustering analysis using the heatmap package v1.0.12 in R employing survival status as the clustering variable. To evaluate for enrichment of differentially methylated genes among pathways, pathway analysis was conducting using two complementary and overlapping annotations: gene ontology (GO [25]) and Kyoto Encyclopedia of Genes and Genomes (KEGG [26]). Pathway analysis, specifically overrepresentation analysis, was pursued using KEGG and GO annotations was performed using clusterProfiler v3.16.1 in R [27], with non-significant differentially expressed genes specified as the “background universe” and accounting for multiple testing using Bonferroni correction. For overrepresentation analysis employing GO annotations, pathways were categorized further into biological process, molecular function, and cellular compartment. Differentially methylated pathways were evaluated in relation to each other and contributing differentially methylated sites by two visualizations of functional enrichment (i.e., dot plot and gene-concept networks) using the enrichplot package v1.8.1 in R.

### TCGA Illumina RNA sequencing analysis

We sought to correlate the expression of genes that harbored differentially methylated sites associated with survival status. We performed an analysis of gene expression collected by RNA sequencing (RNAseq) from early-stage OSCC patients in the TCGA database. Raw gene counts were obtained from TCGA. Only genes with at least 10 counts in at least 90% of the sample were retained for analysis. The Ensembl identifiers (ID) of the gene counts were annotated to Entrez IDs using the EnrichmentBrowser v 2.18.2 Package in R [28]. Annotations for the genes was given using the Homo.sapiens v.1.3.1 package [29]. Correlation of RNAseq gene counts to CpG site methylation was performed using STATA/SE 14.2 (StataCorp, College Station, TX).

### Statistical analyses

Statistical analyses were performed in STATA/SE 14.2. For each cohort, univariate analyses were performed to determine distributional characteristics and assess for randomness of the missing data (variables to be included in the final prognostic panel risk factor score had less than 5% missing values so imputation was not performed). Bivariate analyses with the primary outcome (vital status [survival vs. death] at 5-year follow-up) were performed on candidate variables (based on selection of the investigators from a detailed screening of relevant clinical and demographic risk factors) with the outcome variable. For continuous variables, cut-offs were derived using the chi-square interaction detected by manual adjustment to ensure that cut-offs made sense clinically. Recursive partitioning was used to derive a final non-molecular scoring system to predict survival status at 5-year follow-up with the goal of minimizing the number of misclassified values in the final cell while maximizing the simplicity of the score. Odds ratios at each decision node were rounded to the nearest integer to create the score. Operating characteristics of the derived risk score were calculated on both the discovery (internal cohort, *n* = 515) and validation (TCGA, *n* = 58) cohorts. The concordance statistic (c-index), equivalent to the area under the receiver operating curve (AUROC), was used to assess model discrimination and fit using the derived risk factor score to predict OSCC patients at risk for early mortality and morbidity [30]. The range of the c-index is from 0.5 (random concordance) to 1 (perfect concordance).

The DNA methylation-based, molecular component of the REASON score was performed according to a methylation state transition matrix [31]. For each of the CpG sites, a β-value of < 0.3 indicated an unmethylated state, 0.33–0.75 a hemi-methylated state and > 0.75 a fully methylated state. A gene was considered to be hypermethylated if the methylation level moved from a less methylated state to a more methylated state. Conversely, a gene was considered hypomethylated if there was a state change to a lower level. A change in methylation that did not have a state change was not considered significant [31]. The REASON score was established by combining for the presence or absence of each non-molecular and molecular risk factor. The c-index was derived as described above by comparing the observed survival status at 5 years with the predicted survival status at 5 years using the individual REASON score.

## Results

### Patient cohort characteristics

Our internal cohort of 515 patients and TCGA cohort of 58 patients consisted of patients with early stage (I or II) OSCC based on their pathologic TNM classification. Table 1 details their demographic and clinicopathologic characteristics. The TCGA cohort was 60% male, 93% white, and had a mean age of 64. The majority of patients (68%) were current or previous smokers and 61% of patients used alcohol. Tumor sub-sites included the oral tongue, alveolar ridge, buccal mucosa, or floor of mouth; 57% of the TCGA cohort consisted of oral tongue SCC, with the remainder distributed amongst other sub-sites. With regard to pathologic staging, 31% were stage I and 69% were stage II. In terms of tumor grade, 19% had well-differentiated tumors, with the remaining 81% either moderately or poorly differentiated tumors. PNI was present in 35%, LVI in 6.9%, and positive or close margins in 21% of cases. Five-year survival was 86%. The significant differences between the TCGA cohort and internal cohort are listed in Table 1. Gender, age, self-reported race, and tobacco use were not different between the two cohorts. The internal cohort was 53% male, 93% white, had a mean age of 65, and had 50% current or previous smokers. The internal cohort featured a greater proportion of patients who self-reported Hispanic ethnicity (22% vs 3.6%, *p* = 0.001). The internal cohort had significantly fewer patients that used alcohol (40% vs 61%, *p* = 0.002). There were significant differences in tumor location; while the proportion of patients with tongue SCC was the same in both cohorts (57%), the internal cohort had a higher percentage of alveolar (gingival) SCC than the TCGA cohort (17% vs 5%, *p* < 0.001). There were also differences in tumor grade, with a higher percentage in the internal cohort having well-differentiated tumors (40% vs 19%, *p* = 0.001). A lower percentage in the internal cohort had PNI compared to the TCGA cohort (11% vs 35%, *p* < 0.001). Along the same lines, there were also significantly more patients with a lower pathologic stage in the internal cohort (64% vs 31%, *p* < 0.001). Despite having earlier-stage, more well-differentiated tumors with lower PNI, the risk of death was significantly higher in the internal cohort (37% vs 14%, *p* = 0.001).

**Table 1:**
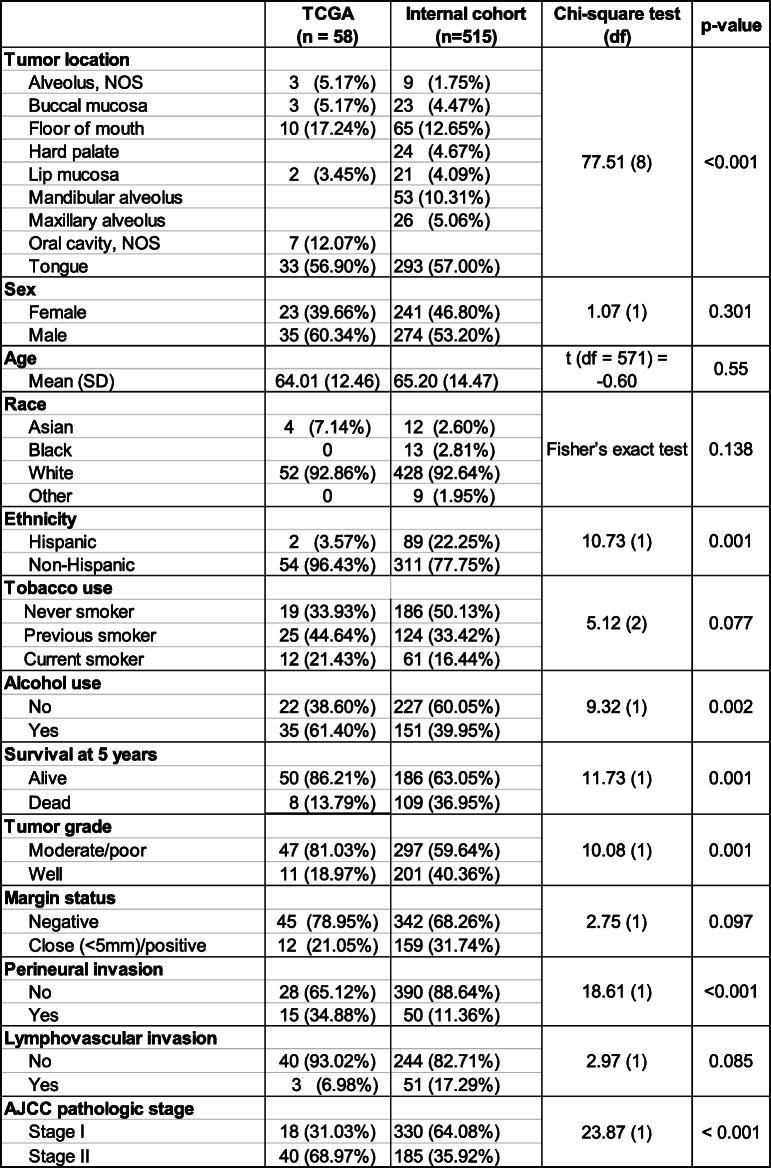
Patient demographics and clinicopathologic characteristics. The table details the characteristics of the two cohorts. Statistical tests and *p*-values are indicated

Abbreviations: *AJCC* American Joint Committee on Cancer, *NOS* Not otherwise specified, *SD* Standard deviation, *TCGA* The Cancer Genome Atlas

### Non-molecular clinicopathologic risk factors

We calculated the c-index using different clinicopathologic factors. The clinicopathologic features with the highest predictive ability among the two cohorts were age, race, sex, tobacco use, alcohol use, histologic grade, stage, PNI, LVI and margin status. This panel of 10 non-molecular features predicted 5-year mortality risk with a c-index = 0.72 for the TCGA cohort, c-index = 0.66 for the internal cohort. Despite the reported differences in clinicopathologic characteristics between the two groups, there were no significant differences in prognostic performance. The two groups combined had a c-index = 0.67 in predicting 5-year mortality. The low c-index is consistent with previous clinical and biomarker studies [4], which have demonstrated that clinicopathologic factors alone could not sufficiently assess disease risk as defined by a c-index of ≥ 0.8. In our current clinical practice, we rely solely on these clinicopathologic factors for risk assessment and treatment decisions.

### Methylation array analysis reveals differentially methylated genes in early stage OSCC patients who did not survive to five years

**Fig. 2:**
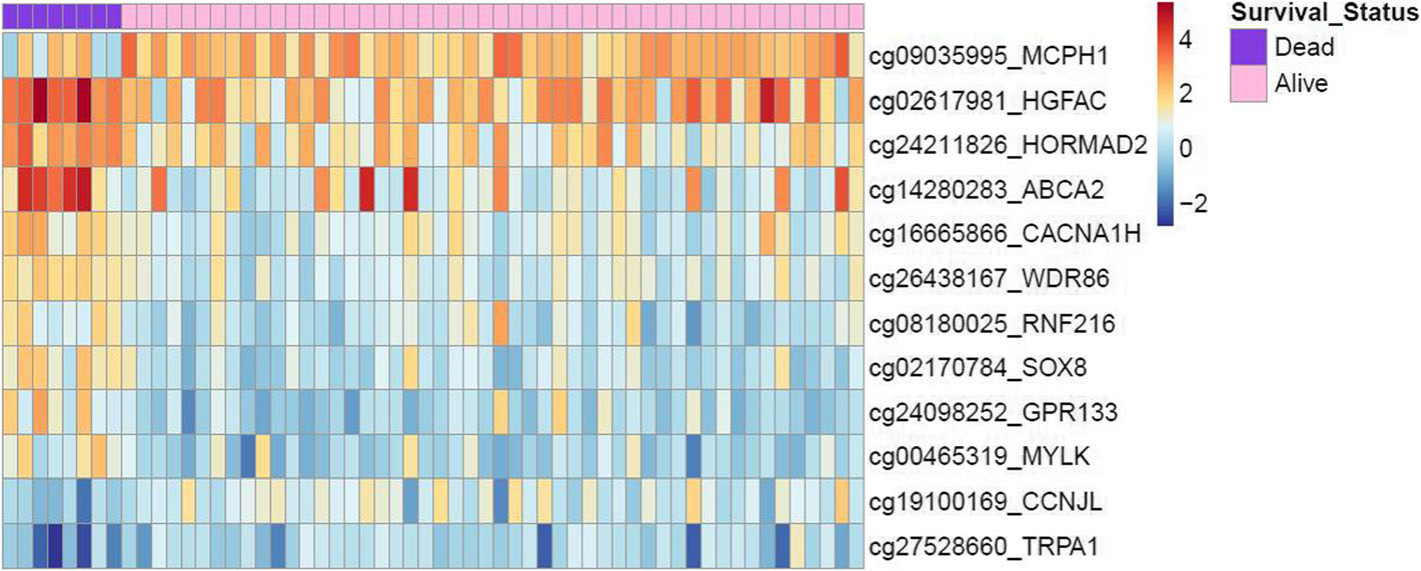
Heat map and hierarchical clustering of differentially methylated genes demonstrate a distinct methylation signature in high-risk vs. low-risk OSCC patients. The figure represents a heat map of the 12 top differentially methylated genes between patients who survived to 5 years vs. those that died in the TCGA cohort. ABCA2 = ATP-binding cassette sub-family A member 2; CACNA1H = calcium voltage-gated channel subunit alpha1 H; CCNJL = cyclin J-like protein; GPR133 = adhesion G protein-coupled receptor D1; HGFAC = hepatocyte growth factor activator; HORMAD2 = HORMA domain containing 2; MCPH1 = microcephalin 1; MYLK = myosin light chain kinase; RNF216 = ring finger protein 216; SOX8 = SRY-box transcription factor 8; TRPA1 = transient receptor potential cation channel subfamily A member 1; WDR86 = WD repeat domain 86

Of the 4544 genes harboring CpG sites meeting criteria for analysis, 12 genes showed an adjusted *p*-value of < 0.1 (Table 2). They included *ABCA2, CACNA1H, CCNJL, GPR133, HGFAC, HORMAD2, MCPH1, MYLK, RNF216, SOX8, TRPA1, and WDR86.* Figure 2 illustrates the methylation state for each of the 58 TCGA patient samples of the 12 top differentially methylated genes using a heat map. Patients who died by 5 years from their cancer are grouped on the left of the heat map, with significant differences in methylation signatures compared to patients who survived to 5 years.

**Table 2:**
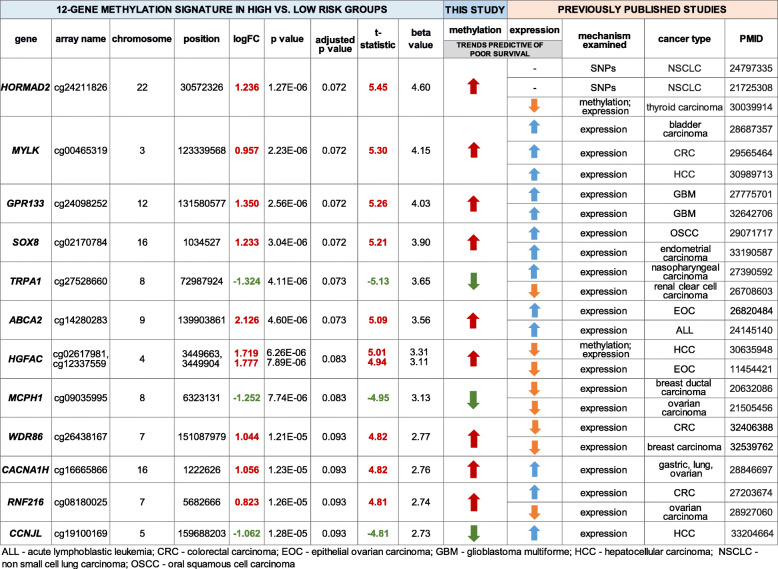
Twelve-gene methylation signature. Gene position and methylation fold-change values are shown. The methylation trends for each gene that are predictive of poor survival in our study are shown, in comparison to the gene expression trends that are predictive of poor survival in previous studies. The PMID of the referenced study is included

*ALL* Acute lymphoblastic leukemia, *CRC* Colorectal carcinoma, *EOC* Epithelial ovarian carcinoma, *GBM* Glioblastoma multiforme, *HCC* Hepatocellular carcinoma, *NSCLC* Non small cell lung carcinoma, *OSCC* Oral squamous cell carcinoma

### Prognostic ability of the REASON score

The REASON score was calculated by combining the 10-factor non-molecular panel with the 12-gene methylation panel composed of 13 CpGs, in which methylation status of each gene was determined using the methylation state transition matrix. The REASON score predicted 5-year disease-specific mortality with a c-index = 0.915.

### Top twelve differentially methylated genes linked to survival in other cancers

A literature search of each of the 12 genes revealed that with the exception of *SOX8,* none of the genes had previously been linked to OSCC in either human or preclinical studies. While many of the genes have not been extensively investigated in preclinical studies to comprehensively map their downstream mechanisms, all 12 genes have at least one publication that links the gene to poor survival in cancer patients. In Table 2 each of the genes is linked to the referenced clinical studies demonstrating poor cancer survival. *HORMAD2* dysregulation through either SNPs [32, 33] or hypermethylation [34] is attributed to poor survival in non-small cell lung cancer (NSCLC) and thyroid carcinoma. *MYLK* over-expression is linked to poor survival in bladder carcinoma [35], colorectal carcinoma [36], and hepatocellular carcinoma [37]. *GPR133* expression is inversely correlated with survival in patients with glioblastoma multiforme [38, 39]. The role of *SOX8* has been already been investigated using in vitro and in vivo models*,* as well as in clinical samples of OSCC. In a clinical study, *SOX8* is over-expressed in chemoresistant patients with tongue SCC and is associated with higher lymph node metastasis, advanced tumor stage and shorter overall survival [40]. Similarly, higher *SOX8* expression is linked to a high tumor histological grade, lymph node metastasis, and shorter overall survival in patients with endometrial carcinoma [41]. *TRPA1* expression in cancer is controversial, with gene over-expression linked to poor survival in nasopharyngeal carcinoma [42] and gene under-expression linked to poor survival in renal clear cell carcinoma [43]. However, a study using International Cancer Genome Consortium data shows that the *TRP* family of genes has varying expression across different cancer types, and that some *TRP* genes have stronger prognostic ability than others [43]. *ABCA2,* which encodes for a membrane-associated protein of the superfamily of ATP-binding cassette transporters, is over-expressed in epithelial ovarian carcinoma and acute lymphoblastic leukemia patients with poor survival [44, 45]. *HGFAC* expression is directly correlated to survival in breast ductal carcinoma and ovarian carcinoma [46, 47]. *WDR86* expression is linked to poor survival in colorectal carcinoma and breast carcinoma [48, 49]. In a clinical study of solid tumors including gastric, lung and ovarian cancer, expression of T-type calcium channel genes including *CACNA1H* is used as a prognostic signature for survival [50]. *RNF216* expression is associated with poor survival in colorectal cancer and ovarian carcinoma, although whether over- or under-expression decreases survival is unknown [51, 52]. *CCNJL* expression is inversely correlated with survival in hepatocellular carcinoma [53]. Of note, differential methylation of the 12 genes has not previously been linked to poor survival in any type of cancer. With the exception of *HORMAD2* and *HGFAC,* published studies on these candidate genes have focused on differential gene expression rather than methylation.

### Functional analysis of the differentially methylated genes

Gene expression data was available for 55 of the 58 TCGA OSCC patients with DNA methylation data. As is becoming increasingly appreciated, gene hypermethylation can result in decreased or increased gene expression [54], which was observed in the TCGA sample. Significant correlation between gene expression and DNA methylation at each gene was observed for 6 (*ABCA2* [*r* = 0.46, *p* = 0.0005], *GPR133* [*r* = 0.42, *p* = 0.0015], *MCPH1* [*r* = 0.31, *p* = 0.024], *RNF216* [*r* = − 0.38, *p* = 0.0045], *TRPA1* [*r* = − 0.60, *p* < 0.0001], *WDR86* [*r* = 0.36, *p* = 0.0072]) of the 12 genes.

**Fig. 3:**
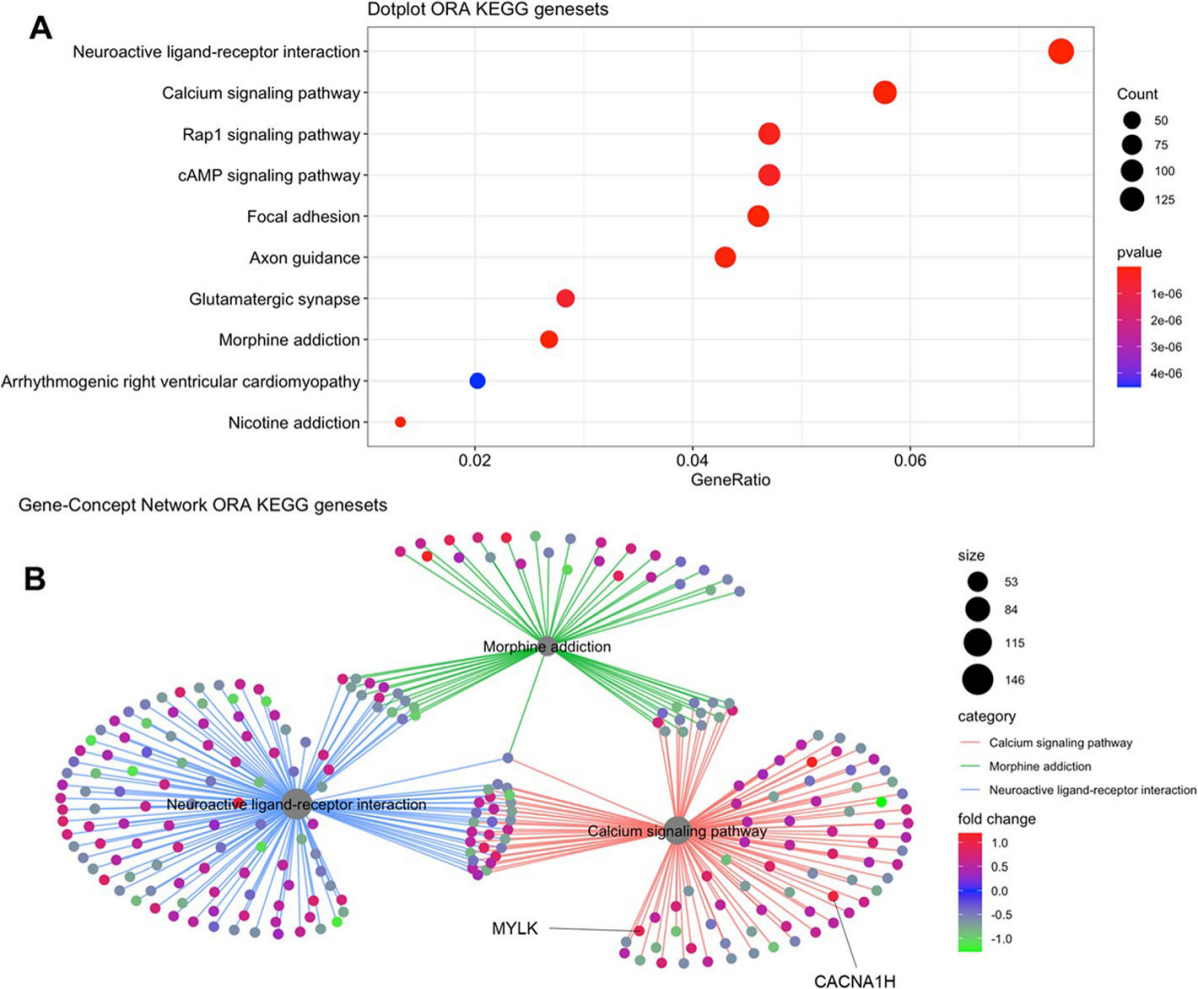
Functional network analysis mapping. Functional enrichment analysis identifies the aggregation of differentially methylated genes onto pathways that aggregate to three concepts. **a** Dot plot of differentially enriched genes that map to the top ten most differentially perturbed methylated pathways (p_adjusted_ < 0.05). **b** The top 3 most statistically differentially methylated pathways are identified by a circle in grey and the fold change in differential methylation of component genes is rendered in color ranging from negative (green) to positive (red) fold change for each gene. The size of each circle is based on the number of genes

We performed gene network analysis through publicly available databases to determine whether our 12 candidate genes were directly involved in established signaling networks. Table 3 details the KEGG pathways that are linked to the candidate genes; Supplemental Table 1 details the GO pathways that are linked to the candidate genes aggregated by gene ontology category (i.e., biological process, cellular compartment, molecular function. Seven of the 12 differentially methylated genes (i.e., *ABCA2, CACNA1H, MCPH1, MYLK, RNF216, SOX8, TRPA1*) mapped to statistically significant differentially methylated pathways. The complex associations between differentially methylated genes mapped to multiple related differentially methylated pathways were visualized using a geneset enrichment dotplot and a gene-concept network plot (Fig. 3). *CACNA1H* and *MYLK* mapped to 5 of the 19 statistically differentially methylated pathways (p_adjusted_ < 0.05; Table 3). The number of differentially methylated genes (*p* < 0.05) among the top ten most differentially methylated pathways (p_adjusted_ < 0.05) are visualized in Fig. 3a. Two (*CACNA1H*, *MYLK*) of the twelve differentially methylated genes included in the REASON score map to the top 3 most differentially methylated pathways: neuroactive ligand-receptor interaction, morphine addiction, and calcium signaling pathways (Fig. 3b).

**Table 3:**
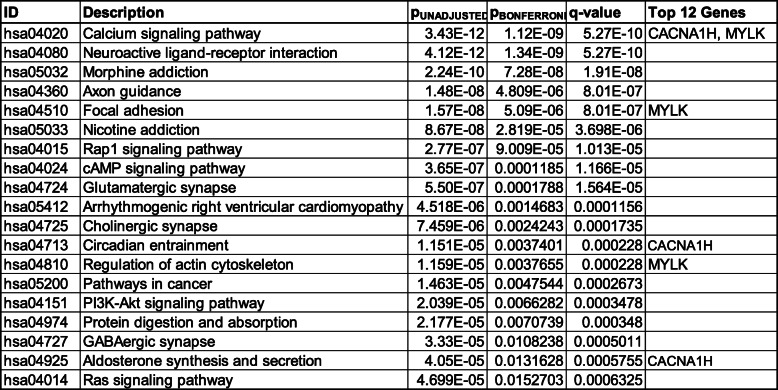
Functional network analysis (KEGG). Differentially methylated pathways (p_adjusted_ < 0.05) based on KEGG annotations are shown. Pathways that include any of the 12 differentially methylated genes included in the prognostic panel are identified

## Discussion

### The REASON score has high accuracy in predicting poor survival of early-stage OSCC

The REASON score that we have constructed in this study relies on non-molecular clinicopathologic factors that are already used in standard clinical practice to assess risk, as well as a 12-gene methylation signature. Previous methylation studies in OSCC have not identified any of these 12 genes as having prognostic power. With the exception of *SOX8*, the genes within the panel have not previously been associated with OSCC. However, while some of these genes have not been firmly established as playing crucial roles in carcinogenesis, all 12 genes have already been linked to cancer survival in genetic association studies on patient tissues [31, 32, 34–42, 45, 46, 48, 50–52]. We therefore believe that the REASON score containing these 12 genes is a promising prognostic tool warranting further validation. The results from this pilot study set the stage for a large scale biomarker validation study in which we will characterize the methylation signatures of our internal cohort, which to our knowledge represents the largest cohort of early-stage OSCC assembled to date. Any gene pathway involved in carcinogenesis undergoes extensive in vitro and in vivo investigations, but a direct link to poor survival in patients requires large clinical cohorts; therefore, to have all 12 genes in the panel already linked to cancer survival in other cancers, is both provocative and rare.

### OSCC patients have poor survival despite rapid advances in cancer treatment

Completion of the human genome project in 2001 ushered in an era of personalized medicine. The hope was that the genetic code would facilitate the development of highly effective biomarker panels to determine risk in cancer patients, and lead to the discovery of anti-cancer drugs that directly target highly dysregulated pathways. Some cancers have seen significantly improved survival as a result of personalized medicine. For example, commercially available genomic tests predict the risk of recurrence in breast cancer patients and are currently used in clinical practice to guide treatment decisions. These biomarker panels were developed from large DNA microarray studies performed in the early 2000s [19, 55]. Currently the most heavily marketed multigene assays include Oncotype DX, which is supported by level II evidence and endorsed by the American Society of Clinical Oncology [20], and Mammaprint, which is supported by level III evidence. These and other advances in breast cancer treatment fueled by in-depth genomic analyses have resulted in a significant improvement in survival over the past two decades, particularly in young women with metastatic disease [56]. However, advances in molecular profiling techniques have not yet led to improved outcomes or clinically useful biomarkers for risk stratification for OSCC. In fact, worldwide OSCC incidence is on the rise [1]. In an epidemiologic study of 22 cancer registries worldwide, tongue cancer incidence has increased in young women < 45 years old without traditional risk factors of tobacco or alcohol use. Despite advances in technology, such as Intensity-Modulated Radiation Therapy (IMRT) for the precise delivery of radiation, improvement in OSCC survival over the past few decades has been modest [3]. Targeted therapies such as cetuximab have demonstrated improved in survival when used with an intensive chemotherapy combination for recurrent or metastatic head and neck SCC (HNSCC), but have yet to show improved outcomes in the definitive setting [57]. Immunotherapy has emerged as a fourth treatment modality for many cancers, and there are now two immune checkpoint inhibitors, nivolumab and pembrolizumab, that are approved for use in HNSCC. Unfortunately, immunotherapy is only effective in 12–20% of HNSCC and their use is currently limited to the recurrent or metastatic setting. Furthermore, the anti-cancer activity seems to require a pre-existing immune infiltrate within the tumor microenvironment, which unfortunately is insufficient or largely absent in most OSCC [58, 59]. For these reasons OSCC patients continue to have poor survival despite recent advances in head and neck cancer treatment.

### OSCC biomarker studies have attempted to predict risk of neck metastasis, but have not generated a clinically viable risk score

Head and neck cancer researchers have attempted to develop a multigene risk score to better tailor treatment for OSCC patients. Studies so far have used differential gene expression, gene amplification and deletions, methylation, and microRNA (miRNA) as potential biomarkers. In contrast to our current study, which identifies high risk patients that might benefit from treatment escalation, the majority of studies have largely focused on preventing over-treatment by developing a biomarker to predict risk of neck metastasis. 20% or more of these patients have occult (i.e.*,* non-detectable by clinical exam or imaging) neck metastasis. Numerous publications, including computational modeling studies [60], retrospective studies [61], and one large prospective clinical trial that compares early stage OSCC patients who receive a prophylactic neck lymphadenectomy to those managed with a watch-and-wait approach [62], all demonstrate that the > 20% risk of occult metastasis portends a poor survival in the absence of a prophylactic neck lymphadenectomy. As a result, it is standard of care for early stage OSCC patients to receive a prophylactic neck lymphadenectomy, even if this practice involves over-treatment for up to 80% of patients with concomitant morbidity, including shoulder dysfunction, nerve damage and lymphedema [63]. This clinical practice necessitates a need to develop a more nuanced approach of risk stratifying patients. However, to date no molecular signature exists that predicts risk of neck metastasis with high enough accuracy for use in a clinical setting. Earlier biomarker studies performed in the early 2000s used custom-built in-house DNA microarray gene expression profiling to discover differentially expressed genes between patients with and without neck metastasis. Roepman et al. from the Netherlands identified a 102-gene signature to predict neck metastasis [64, 65]. The signature was 77% accurate in predicting presence of neck metastasis and 100% accurate in predicting lack of neck metastasis, with an overall accuracy of 86%. The authors proceeded to validate their signature 7 years after the initial study, after switching their platform to a commercially available microarray that was CLIA/ISO-approvable. In the multi-center validation of 222 patients comprised of OSCC and oropharyngeal SCC (OPSCC), the authors determined that their gene expression signature had a negative predictive value (NPV) of 72% for all stages of OSCC and OPSCC, which was increased to 89% in 101 early stage (I/II) OSCC patients alone [63]. In a clinical decision model that incorporated the gene signature, the authors predicted that they would be able to avoid over-treatment with a neck dissection in 32 of the 101 early stage OSCC patients [63]. The improved NPV when only considering early stage I/II OSCC patients alone underscores the importance of recognizing that OSCC is a disease subset that is distinct from all other head and neck cancer sub-sites.

Focusing instead on gene amplifications and deletions, Albertson et al. used array comparative genomic hybridization (CGH) [66] to identify dysregulated pathways in OSCC. Their group was the first to identify dysregulation of the Notch signaling pathway in OSCC. This finding was later validated in a study by Grandis et al. that defined the mutational landscape of head and neck cancer [67]. The group went on to define a biomarker of chromosomal aberrations that included +3q24-qter, −8pter-p23.1, +8q12-q24.2, and + 20, which distinguished a major subgroup (70–80% of OSCC patients, termed 3q8pq20 subtype) from the remainder (20–30% of OSCC patients, non-3q8pq20). The non-3q8pq20 biomarker had a high negative predictive value (0.93–1.0), but low positive predictive value (0.46–0.77) [68]. This biomarker has not yet been validated in a clinical trial. Taken together, the biomarker trials to date focused on predicting the risk of neck metastasis have had modest success. If a robust biomarker could be validated, it would be highly beneficial to surgeons and patients alike, as it will allow them to make an informed decision on the need for an elective neck dissection, while avoiding over-treatment.

### There is a need for biomarkers to predict poor survival in early stage OSCC

Rather than focusing on biomarkers to de-escalate neck dissections, other investigators have taken a similar approach to our current study and focused instead on developing biomarkers of poor survival in OSCC patients, with the intent of identifying high risk patients that might benefit from treatment escalation. Chauhan et al. developed a biomarker panel of 5 proteins qualitatively assessed with immunohistochemistry to predict mortality in OSCC patients. The test and validation cohorts included both early- and late-stage OSCC patients (stages I-IV). Their validated biomarker panel had a c-index = 0.69 [69]. Yoon et al. developed a microRNA (miRNA)-based scoring system that combined clinicopathologic data with miRNA signatures. They focused only on early-stage OSCC patients. The authors discovered and validated this prognostic panel using 568 early stage (I/II) OSCC patients with known 5-year survival outcome. They examined multiple clinicopathologic factors, including TNM classification, histologic grade, PNI, LVI, depth of invasion, margin status, race, smoking and alcohol use, along with 2083 miRNAs.

Clinicopathologic data alone only predicted risk of death at 5 years with an accuracy of c-index = 0.672 (*p* < 0.001). Their top 3 candidate miRNAs were miRNA-127-3p, miRNA-655-3p, and miRNA-4736 (c-index = 0.810; *p* < 0.001). Based on c-index calculations they determined that a 5-plex panel consisting of TNM classification, histologic grade, miRNA-127-3p, miRNA-655-3p, and miRNA-4736 had the highest prognostic power in predicting risk of death at 5 years, with a c-index = 0.832 (*p* < 0.001) [4]. While our clinical cohort includes a proportion of the cohort used by Yoon et al.*,* analysis of our entire cohort has yielded different significant clinicopathologic factors, which we combined with our methylation signature to produce the REASON score.

The REASON score developed in this study predicts risk of death by 5 years in early stage OSCC patients with a c-index of 0.915. The risk score was developed by leveraging both a large internal cohort with publicly available TCGA data, focusing specifically on oral cavity sub-sites to maximize the likelihood of discovering meaningful biomarkers in a highly capricious disease. Due to the modest sample size available for the initial development of the molecular portion of the REASON score (TCGA), cross-validation approaches (e.g., k-fold or leave-one-out methods) would not be valid nor appropriate as possible bias would be introduced due to the generation of performance estimates on smaller subsets of the current data set size [70]. An adequately large data set will be required to permit testing, training and validation approaches to be applied to the preliminary REASON score. We are currently validating the REASON score by performing methylation array analysis in our entire internal cohort. Additionally, we are enrolling OSCC patients in a prospective study that will validate the REASON score using non-invasive brush biopsies.

## Conclusions

In this study we used an internal cohort and publicly available cohort to derive salient clinicopathologic factors and combined them with a 12-gene methylation signature to create the composite molecular/non-molecular REASON score, which had high prognostic performance in identifying early-stage (I/II) OSCC patients with high risk of death in 5 years.

## Supplementary Information

**Additional file 1: Table S1.** Functional network analysis (GO). Differentially methylated pathways (adjusted *p*-value< 0.05) based on GO annotations are shown. Differentially methylated pathways were evaluated based on Biological Process (BP), Molecular Function (MF), and Cellular Compartment (CC) ontologies. Pathways that include any of the 12 differentially methylated genes included in the prognostic panel are identified.

## Data Availability

All data generated for this study are included in this article.
